# Effects of digital health interventions on self-care and quality of life in patients with an ostomy: A systematic review and meta-analysis

**DOI:** 10.1016/j.apjon.2026.100955

**Published:** 2026-04-16

**Authors:** Donglin Wang, Na Zhou, Yingjie Zhang, Jianjuan Dai

**Affiliations:** a General Surgery Department, Zhoushan Hospital, Zhoushan, China; b Nursing Department, Sir Run Run Shaw Hospital, School of Medicine, Zhejiang University, Hangzhou, China; c General Surgery Department, Zhoushan Hospital, Zhoushan, China

**Keywords:** Ostomy, Digital health, Telehealth, Mobile applications, Quality of life, Systematic review

## Abstract

**Objective:**

This study aimed to systematically evaluate the impact of DHIs on self-management, quality of life (QoL), and clinical outcomes in patients with an ostomy.

**Methods:**

We conducted a systematic review and meta-analysis of randomized controlled trials (RCTs). Fourteen RCTs (*n* = 1753) were included. Primary outcomes were self-management competence and QoL; secondary outcomes included stoma complications, psychological well-being, patient satisfaction, and health care utilization. Data were pooled using random-effects models and certainty of evidence was assessed via Grading of Recommendations Assessment, Development and Evaluation (GRADE).

**Results:**

DHIs significantly improved self-management competence (SMD = 0.96, 95% CI: 0.65–1.23) and QoL (SMD = 0.67, 95% CI: 0.30–1.03). They also reduced the risk of stoma-related complications by 46% (RR = 0.54, 95% CI: 0.35–0.84). Benefits for psychological well-being and patient satisfaction were significant but associated with high heterogeneity and low certainty. Subgroup analyses revealed that effects were substantially larger in upper-middle-income countries and among patients with permanent stomas. Exploratory analyses found no significant difference in unplanned readmissions or patient retention between DHI and control groups.

**Conclusions:**

DHIs are effective for improving self-care, QoL, and reducing complications in patients with an ostomy. The greatest impact was observed in resource-limited settings and for permanent diversions. These tools should be integrated into standard postoperative care pathways. Future research requires standardized outcome measurement and long-term evaluations to confirm sustainability.

## Introduction

Ostomy formation is a life-saving surgical procedure that creates an abdominal stoma to divert feces or urine. It is an essential management strategy for a range of gastrointestinal and urological conditions, including colorectal cancer (CRC) and inflammatory bowel disease.^1^ The procedure may be temporary or permanent and most frequently takes the form of a colostomy, ileostomy, or urostomy.^2^ Temporary ileostomies pose particular management challenges due to high-output effluent and an increased risk of peristomal skin injury.^3^ Although stoma creation can be life-preserving, many patients experience substantial postoperative morbidity and long-term impairment in daily functioning. Patients also reported complications including dermatitis, leakage, prolapse, retraction, stenosis, bleeding, and parastomal hernia.^4^, ^5^, ^6^ Moreover, complication rates remain high in certain stoma types.^2^

Stoma formation rates vary by region and approximately 130,000 new stomas are created each year.^2^ According to an estimate around 13.5 million individuals worldwide currently live with a stoma.^2^^,^^7^ There are three main types of stoma formation including colostomy, ileostomy, and urostomy. A stoma can also be temporary or permanent depending on the severity of the condition.^4^, ^5^, ^6^ Temporary ileostomies are particularly complex due to their corrosive and high-volume output and skin management difficulties.^8^ CRC and its complications are a leading indication for stoma creation and CRC is the third most common cancer and the second leading cause of cancer-related mortality globally.^3^ In China alone, nearly 1 million people live with a permanent colostomy with about 100,000 new cases annually.^4^

The physical sequelae of stoma formation are compounded by substantial psychological and social burdens Altered body image, anxiety about leakage or odor, social withdrawal, and caregiver strain are widely reported and contribute to reduced quality of life (QoL) among people living with a stoma.^4^, ^5^, ^6^ Improper care frequently leads to a high incidence of complications, as reported in up to 70% of patients.^9^ Effective self-care encompassing peristomal skin care, appliance management, dietary modification, effluent control, and early recognition of complications is therefore central to preserving independence and reducing complications after discharge.^3^^,^^9^^,^^10^ Patients also require mastery of appliance management and timely recognition and management of complications.^3^^,^^11^^,^^12^

Conventional stoma education and follow-up typically rely on intermittent, face-to-face encounters with specialist nurses.^12^ Short hospital stays and geographic barriers limit access to ongoing specialist support after discharge, increasing the potential for preventable complications and readmissions.^6^^,^^13^ Digital health technologies have been proposed as a strategy to address these gaps by delivering remote education, monitoring, and on-demand consultation.^4^ In this review digital health interventions (DHIs) refers to interventions in which the digital modality is the primary vehicle for delivery examples include mobile applications, synchronous or asynchronous telemedicine consultations, web-based educational platforms, and digitally enabled remote monitoring systems.^3^^,^^9^^,^^11^^,^^13^

Many studies have evaluated a range of DHIs for patients with an ostomy, but study designs, intervention modalities, and outcome measures vary widely across studies.^3^^,^^10^^,^^11^^,^^13^, ^14^, ^15^ Reported effects on self-management ability and QoL are inconsistent, and many trials use heterogeneous or non-standardized instruments to measure outcomes.^4^^,^^16^, ^17^, ^18^ These methodological differences complicate interpretation and limit clinical generalizability. A comprehensive and methodologically rigorous synthesis was therefore required to clarify the overall effectiveness of these interventions and to identify factors contributing to heterogeneity in outcomes.

The present systematic review and meta-analysis aimed to synthesize randomized controlled trials assessing DHIs for adult patients with an ostomy. The primary outcomes were self-management competence and quality of life (QoL). Secondary objectives included assessing the impact on clinical outcomes including stoma complications, readmissions, psychological well-being, patient satisfaction. The review aims to provide clinically effective evidence for improving self-management and reducing complications in ostomy care.

## Methods

### Study design

This review was conducted according to a predefined protocol and reported in accordance with Reporting Items for Systematic Review and Meta-Analysis (PRISMA) 2020 reporting guidance^19^ and the Cochrane Handbook for Systematic Reviews of Interventions.^20^ The review was prospectively registered with PROSPERO (CRD420251123171; registration record: https://www.crd.york.ac.uk/PROSPERO/view/CRD420251123171).

The primary objective was to evaluate the effectiveness of DHIs on self-care ability and QoL in adults with an ostomy. Secondary objectives included effects on psychological well-being, hospital readmission, patient satisfaction, and adherence.

### Eligibility criteria

The eligibility criteria for this review were defined using the Population, Intervention, Comparator, and Outcome (PICO) framework.

#### Participants (P)

Randomized controlled trials (RCTs) enrolling adults (≥ 18 years) with an established ostomy were eligible. Trials restricted to pediatric populations (< 18 years) or populations without an ostomy were excluded.

#### Interventions (I)

Digital health interventions were eligible when the digital modality served as the primary vehicle for delivery (defined a priori as delivering ≥ 50% of the intervention content or being required for core intervention components). Eligible modalities included mobile applications, synchronous or asynchronous telemedicine, web-based platforms, and remote digital monitoring systems.

#### Comparators (C)

Eligible comparators included usual care, face-to-face education, or other non-digital interventions. Trials without any comparator group were excluded.

#### Outcomes (O)

Primary outcomes were self-care ability and QoL measured at the longest reported follow-up using validated instruments when available. Studies that used non-validated or self-constructed scales were eligible for inclusion but were identified a priori and subjected to sensitivity analyses excluding those studies. Secondary outcomes included psychological well-being, readmission rates, patient satisfaction, and adherence.

#### Study designs and time window

Only peer-reviewed RCTs were included. Studies published in English or Chinese between 2015 and 2025 were eligible. The last search date is 01-07-25.

### Search strategy

A comprehensive search was conducted in six electronic databases from database inception to 01-07-25. The conceptual PICO-based search terms are summarized in Supplementary Table S1. The complete search strings are provided in Supplementary Table S2 (PubMed), Table S3 (Embase), Table S4 CENTRAL, Table S5 CINAHL, and Table S6 (CNKI/Wanfang). Search terms combined controlled vocabulary (MeSH/Emtree) and keywords for ostomy and digital health interventions. Search strategies were adapted to each database's syntax. We screened reference lists of included trials and relevant reviews and contacted study authors when necessary to obtain missing data. Full reproducible search strings for all databases are provided in Supplementary Tables S1-S6.

### Study selection

**Fig. 1:**
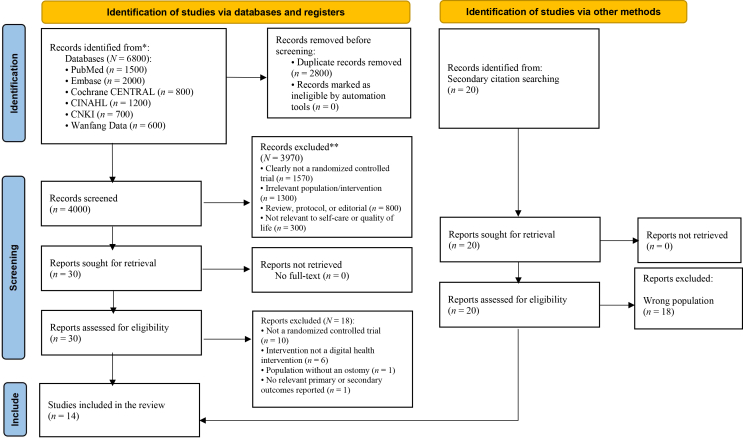
Flow diagram illustrating the original process of screening and identification of studies.∗ Records identified from databases and registers; ∗∗ Records excluded at title/abstract screening; reasons for exclusion included not a randomized controlled trial, irrelevant population/intervention, review/protocol/editorial, and not relevant to self-care or quality of life.

Search results were imported into EndNote X9 and deduplicated. Title/abstract screening and full-text assessment were performed independently by two reviewers after a pilot calibration exercise to ensure consistent application of eligibility criteria. Any citations judged potentially eligible by either reviewer advanced to full text. Discrepancies at either stage were resolved by discussion or adjudication by a third reviewer. Reasons for full-text exclusions are recorded and presented in the PRISMA flow diagram (Fig. 1).

### Data extraction and management

Two reviewers independently extracted data using a standardized form. Extracted data encompassed key study characteristics, intervention and comparator details, outcome measures, and numerical results, along with information necessary for risk-of-bias evaluation and Grading of Recommendations Assessment, Development and Evaluation (GRADE) assessment. We also recorded whether each outcome was prespecified as the trial primary endpoint. Discrepancies were resolved by discussion or third-party adjudication. Extracted data are summarized in Table 1.

**Table 1:** Characteristics of included studies evaluating digital health interventions for patients with an ostomy (*N* = 14).

	Study	Country (Income)^a^	Design	Sample Size (I/C)^b^	Ostomy Type	Intervention Modality (Classification)^c^	Duration	Outcomes / RoB / GRADE^d^
1.	Chen et al. (2016)	China (UMIC)	RCT	60/60	Permanent	Multimodal: Mobile video + WeChat/QQ support	12 weeks	ESCA, QCL / Some concerns / Downgraded^e^
2.	Sun et al. (2016)	China (UMIC)	RCT	40/36	Mixed	mHealth: WeChat care clinic (Photo diagnosis)	4 weeks	Self-care practice, Satisfaction / High / High RoB
3.	Su et al. (2019)	China (UMIC)	Multicenter RCT	50/57	Temporary	Multimodal: Care bundle (manual + phone)	12 weeks	SSES, Stoma-QOL / Low / Multicenter strength
4.	Sun et al. (2019)	China (UMIC)	RCT	30/30	Temporary	mHealth: “317 Hu” App + WeChat interaction	4 weeks	CPCKAPS, OAI / High / Downgraded^f^
5.	Wang et al. (2019)	China (UMIC)	RCT	21/20	Urinary	Multimodal: Nurse-led + Video/Phone	24 weeks	Skin self-care, QoL / High / Downgraded^e^
6.	Augestad et al. (2020)	Norway (HIC)	RCT	52/58	Mixed	Telemedicine: Synchronous video consultations	52 weeks	Baxter, EQ-5D / Low / Long-term follow-up
7.	Taylan & Aksoy (2021)	Turkey (UMIC)	RCT	30/30	Mixed	Tele-nursing: Structured telephone counseling	10 weeks	OAI, AQLS / Some concerns / Downgraded^f^
8.	Ambe et al. (2023)	Germany (HIC)	Crossover RCT	139/139	Mixed	mHealth/Sensor: Wearable + App tracking	8 weeks	WHODAS, OLI / Low / Validated disability metric
9.	Hao et al. (2023)	China (UMIC)	RCT	50/50	Mixed	Virtual platform: Online lectures + WeChat	12 weeks	SSES, SF-36 / Some concerns / Possible performance bias
10.	Ko et al. (2023)	Taiwan (HIC)	RCT	54/54	Mixed	Multimedia: Education program (CD-ROM)	12 weeks	Self-care, Stoma-QOL / Some concerns
11.	Xu et al. (2023)	USA (HIC)	Pilot RCT	12/5	Mixed	mHealth + Wearables: App + Fitbit + telehealth	8.5 weeks	FACT-G / High / Exploratory results
12.	Ding et al. (2024)	China (UMIC)	RCT	144/151	Urinary	Social Media: Peer-led WeChat groups	24 weeks	KAP, WHOQOL-100 / Low / Large sample size
13.	Krouse et al. (2024)	USA (HIC)	RCT	80/93	Mixed	Telehealth: Peer-coaching + Video	24 weeks	PAM, COH-QOL-O / Low / High quality
14.	Storm et al. (2024)	Netherlands (HIC)	RCT	96/112	Mixed	mHealth (App): Stoma App (Interactive)	12 weeks	Psychosocial, Stoma-QOL / Low / Technical issues noted

C, control group; COH-QOL-O, City of Hope Quality of Life-Ostomy; ESCA, Exercise of Self-Care Agency; HIC, high-income country; I, intervention group; KAP, Knowledge, Attitude and Practice; OAI, Ostomy Adjustment Inventory; PAM, Patient Activation Measure; QoL, quality of life; RCT, randomized controlled trial; RoB, Risk of Bias (RoB 2.0); SC, self-care; SSES, Stoma Self-Efficacy Scale; UMIC, upper-middle-income country.

a Economic classification based on the World Bank definition at the time of study publication.

b Sample size analyzed (Intervention / Control).

c Modality Classification: Interventions were classified as Digital-Primary (e.g., mHealth, Virtual Platform, Telemedicine) or Multimodal/Adjunctive where digital tools supported a predominantly in-person or manual-based nursing intervention.

d Indicates a non-validated or binary outcome measure used as a proxy for self-care ability.

e Overall risk of bias judgment derived from the Cochrane RoB 2.0 tool.

f GRADE Factors: Notes on study design, directness, precision, or measurement issues that influenced the certainty of evidence assessment.

### Risk of bias assessment

**Fig. 2:**
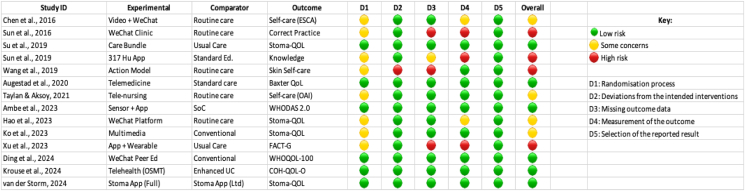
Risk of Bias Assessment for Included Studies (RoB 2.0). Risk of bias was evaluated for 14 randomized controlled trials using the Cochrane Risk of Bias 2.0 (RoB 2.0) tool. Domains assessed were: D1, randomization process; D2, deviations from intended interventions; D3, missing outcome data; D4, measurement of the outcome; and D5, selection of the reported result. Color coding: green (low risk), yellow (some concerns), and red (high risk). COH-QOL-O, City of Hope Quality of Life–Ostomy; ESCA, Exercise of Self-Care Agency; FACT-G, Functional Assessment of Cancer Therapy-General; OAI, Ostomy Adjustment Inventory; SoC/UC, standard of care/usual care; Stoma-QOL, Stoma-specific Quality of Life. (For interpretation of the references to colour in this figure legend, the reader is referred to the web version of this article.)

Risk of bias for each included RCT was assessed independently by two reviewers using the Cochrane Risk of Bias 2.0 (RoB 2)^21^ tool across its five domains. Each domain received a rating of ‘low risk of bias’, ‘some concerns’, or ‘high risk of bias’. Domain-level judgments informed an overall risk-of-bias judgment per outcome. Discrepancies were resolved by consensus or a third reviewer. RoB 2 assessments are presented in Fig. 2.

### Data synthesis

Data synthesis employed a comprehensive, two-pronged approach that integrated both qualitative and quantitative methods.

#### Qualitative synthesis

A narrative synthesis approach was employed to summarize the findings of all included studies. Quantitative synthesis was performed when at least two independent randomized comparisons reported compatible outcome measures.

#### Quantitative synthesis (meta-analysis)

We calculated standardized mean differences (SMD; Hedges g) with 95% confidence intervals (CIs) for continuous outcomes reported on different scales. We used risk ratios (RRs) with 95% CIs for dichotomous outcomes. The primary meta-analytic approach used a random-effects model with the restricted maximum likelihood (REML) estimator to account for between-study heterogeneity. Between-study heterogeneity was quantified using the *I*^*2*^ and τ^2^ statistics. *I*^*2*^ values were interpreted according to Cochrane thresholds: 0%–40% (low), 30%–60% (moderate), 50%–70% (substantial), and 75%–100% (considerable heterogeneity). The presence of statistical heterogeneity was assessed using the Cochran Q test, with *P* < 0.10 considered statistically significant. All statistical computations were executed using R statistical software (v4.2.2), specifically utilizing the meta and Meta for packages.

#### Handling multiple effect sizes and dependence of data

The primary analyses used one effect size per randomized comparison to avoid double-counting participants. Selection hierarchy for the primary effect per trial was: (1) prespecified primary outcome (validated instrument), (2) validated instrument most commonly used across included trials, (3) longest follow-up measure if multiple timepoints reported. Trials reporting multiple correlated outcomes from the same participants were reduced to a single selected effect per trial.

### Subgroup and sensitivity analyses

Subgroup and sensitivity analyses were conducted to explore potential sources of heterogeneity.

#### Subgroup analyses

Prespecified subgroup analyses examined intervention modality, ostomy type, intervention duration, and country income level. Subgroup meta-analyses were performed only when a subgroup contained at least two independent randomized comparisons. For subgroup strata with a single study we present descriptive results without pooled estimates.

#### Sensitivity analyses

Three sensitivity analyses were performed to evaluate the robustness of the primary findings. First, an outlier-exclusion analysis was conducted by removing trials with extreme effect sizes (SMD > 2.0). Second, a methodological sensitivity analysis was performed by excluding trials that utilized non-validated or self-constructed outcome instruments as these may introduce measurement bias. Third, a leave-one-out (LOO) analysis was executed by iteratively removing one trial at a time to determine if the pooled result was disproportionately influenced by a single study. Publication bias was assessed via visual inspection of funnel plots and Egger's test for outcomes comprising ≥ 10 trials.

### GRADE assessment of evidence certainty

We assessed the certainty of evidence for each primary outcome using GRADE. This systematic framework considers five principal domains that may downgrade evidence certainty: (1) Risk of bias (2) Inconsistency, (3) Indirectness (4) Imprecision and (5) Publication bias. The final certainty of evidence for each key outcome was categorized as ‘high’, ‘moderate’, ‘low’, or ‘very low’. A Summary of Findings table with explicit downgrading rationale is provided (Table 2).

**Table 2:** GRADE evidence profile for digital health interventions for patients with an ostomy.

Outcome	No. of Studies (k /n)	Relative Effect (95% CI)	Certainty of Evidence	Plain Language Summary
Self-management skills	13/(1677)	SMD 0.96 [0.65, 1.23]	Low^a^ ⊕⊕◯◯	DHIs likely improve self-care practice in patients with an ostomy.
Quality of life	13/(1628)	SMD 0.67 [0.30, 1.03]	Low^b^ ⊕⊕◯◯	Patients using DHIs may report higher quality of life than with routine care.
Postoperative complications	7/(1180)	RR 0.54 [0.35, 0.84]	Moderate^c^ ⊕⊕⊕◯	DHIs reduce the risk of clinical complications by approximately 46%.
Psychological well-being	12/(1570)	SMD 0.85 [0.17, 1.54]	Low^a^ ⊕⊕◯◯	DHIs likely improve adaptation and reduce psychological distress.
Patient satisfaction	12/(1048)	SMD 1.42 [0.43, 2.41]	Very low^d^⊕◯◯◯	Satisfaction is high, but the evidence base remains inconsistent.
Unplanned readmissions	3/(577)	RR 0.96 [0.32, 2.85]	Low^e^⊕⊕◯◯	Evidence is insufficient to determine a reduction in readmissions.

CI, confidence interval; DHI, digital health intervention; RR, risk ratio; SMD, standardized mean difference; GRADE, Grading of Recommendations Assessment, Development and Evaluation.

a Certainty reduced due to high statistical heterogeneity ($I^{2}>75\%$) and potential for publication bias.

b Certainty reduced due to serious risk of bias across several included trials.

c Certainty rated moderate as findings remained robust across sensitivity analyses and primary subgroups.

d Certainty reduced due to serious inconsistency and wide confidence intervals (imprecision).

e Certainty reduced due to serious imprecision; the 95% CI includes both benefit and harm.

## Results

### Study selection

The initial systematic search across electronic databases yielded 6800 records and an additional 20 records were identified through manual searches of reference lists and grey literature. Following the removal of 2800 duplicates and 20 records identified as ineligible by automated screening tools and 4000 unique citations underwent title and abstract screening. Of these, 3970 records were excluded based on pre-defined criteria, primarily due to non-randomized study designs (*n* = 1570), irrelevant populations or interventions (*n* = 1300), review/protocol status (*n* = 800), or lack of focus on self-care and QoL (*n* = 300).

Thirty reports were retrieved for full-text eligibility assessment. Eighteen reports were subsequently excluded with documented reasons including ten were not randomized controlled trials, six did not meet the definition of a digital health intervention, one that involved a non-ostomy population, and one that failed to report relevant primary or secondary outcomes. Finally 14 unique RCTs met all eligibility criteria and were included in both the qualitative synthesis and the quantitative meta-analysis. The stepwise selection process is illustrated in the PRISMA 2020 flow diagram (Fig. 1).

### Characteristics of included studies

Of the 14 included studies 7 originated from China^12^^,^^22^, ^23^, ^24^, ^25^, ^26^, ^27^ and two from the USA.^28^^,^^29^ Only one each from Germany,^14^ the Netherlands,^30^ Norway,^15^ Taiwan,^31^ and Turkey.^32^ Thirteen studies employed a parallel-group design and only one utilized the crossover design.^14^ Total sample sizes ranged from 23 to 340 participants encompassing a cumulative total of 2023 patients. Participants were adult patients with new or established ostomies resulting from oncological, inflammatory, or traumatic conditions. Specifically, the cohorts included those with permanent urinary stomas,^27^ colostomies, ileostomies, or mixed ostomy types.^29^

The DHI modalities were heterogeneous, encompassing mobile applications,^30^^,^^32^ WeChat-based applications,^12^^,^^22^ dedicated eHealth platforms,^29^ and telehealth services.^15^^,^^28^ Comparators included standard care, conventional face-to-face education, or modified digital tools. Intervention durations ranged from 1 to 6 months with follow-up assessments primarily conducted within the first 6 months post-intervention. Notably, one study provided a longitudinal follow-up extending to 2 years.^23^ Primary outcomes consistently focused on self-care ability, self-efficacy, and quality of life, assessed via various scales. It should be noted that Wang et al.^27^ and Chen et al.^25^ utilized self-designed or modified general health-related instruments. A detailed summary of these characteristics is provided in Table 1.

### Risk of bias assessment

Risk of bias was assessed using the Cochrane RoB 2 tool (Fig. 2). Overall, 4 studies (28.6%) were rated as high risk^24^^,^^26^^,^^27^^,^^29^ primarily due to concerns regarding missing outcome data (D3) and outcome measurement (D4). Another 4 studies (28.6%) were categorized as having some concerns^11^^,^^22^^,^^31^^,^^32^ frequently originating from the randomization process (D1) or unblinded patient-reported outcomes. The remaining 6 studies (42.8%) were judged to be at low risk of bias.^12^^,^^14^^,^^15^^,^^23^^,^^28^^,^^30^ The most prevalent methodological challenge across the trials was the inability to blind participants and providers which increased the potential for performance and detection bias.

### Meta-analysis (quantitative synthesis)

Considering the anticipated and observed high levels of heterogeneity across studies, SMDs were pooled using random-effects models for continuous outcomes.

#### Self-management competence

**Fig. 3:**
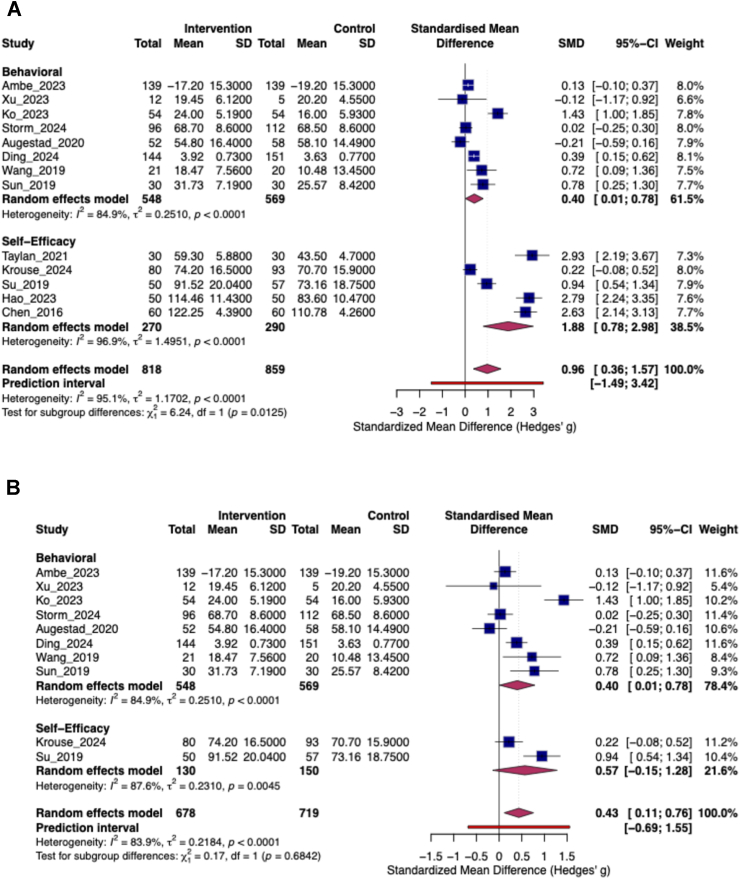
A: Forest plot of digital health interventions versus control for self-management competence. The analysis is stratified by outcome type (Behavioral vs. Self-Efficacy). Squares represent the standardized mean difference (SMD) for individual studies; diamonds represent the pooled SMD; horizontal lines represent 95% CIs. SMD was calculated as Hedges' g to correct for small-sample bias. Fig. 3B: Sensitivity analysis excluding extreme outliers (SMD > 2.0) for self-management competence. This forest plot excludes Chen et al. (2016), Taylan et al. (2021), and Hao et al. (2023) to investigate the source of heterogeneity. The reduction in *I*^2^ from 95.1% to 83.9% indicates that these trials were primary drivers of statistical variance, though the overall treatment effect remains significant.

A meta-analysis of the self-management competence domain comprising 13 RCTs (*n* = 1677) demonstrated a significant positive effect in favor of digital health interventions compared to control groups. Using a random-effects model, the pooled SMD was 0.96 (95% CI: 0.65–1.23; *P* = 0.002), indicating a large beneficial effect. However, substantial heterogeneity was observed across the included studies (Q = 242.65, df = 12, *P* < 0.001; *I*^2^ = 95.1%). Subgroup analysis revealed that the effect was more pronounced in studies measuring self-efficacy (*k* = 5; SMD = 1.88) compared to those measuring behavioral self-care ability (*k* = 8; SMD = 0.40) (Fig. 3A).

Sensitivity analyses provided further nuance to these findings: (1) Outlier exclusion: removal of three extreme outliers^11^^,^^22^^,^^32^ (all SMD > 2.0) reduced the pooled effect size to a more conservative estimate (SMD = 0.43; 95% CI: 0.11–0.76; *P* = 00.008) and decreased heterogeneity to *I*^2^ = 83.9% (Fig. 3B); (2) Instrument validity: Excluding trials that utilized non-validated or self-constructed scales (Chen 2016^25^ and Wang et al.^27^) resulted in a pooled SMD of 0.81 (95% CI: 0.46–1.16; *P* < 0.001). Heterogeneity remained high following this exclusion (*I*^2^ = 91.0%). The overall effect remained significant but statistical significance was lost within the self-efficacy subgroup as indicated in sensitivity analysis excluding studies with non-validated Scales for Self-management. It indicated that measurement instrument selection is a primary driver of perceived confidence-related outcomes (Supplementary Fig. S1). LOO Analysis: The LOO analysis confirmed the stability of the direction of the effect. Across all iterations, the heterogeneity remained substantial with *I*^2^ values ranging from 93.3% to 95.7%. No single trial's removal was sufficient to resolve the overall statistical inconsistency or transform the overall result to non-significance (*P* > 0.05) (Supplementary Fig. S2).

Exploratory subgroup analysis identified economic setting, stoma type, and intervention duration as significant moderators of effect. DHIs demonstrated greater efficacy in upper-middle-income countries (UMICs) compared with high-income countries (HICs) (SMD: 1.58 vs. 0.26; *P* = 0.007) **(**Supplementary Fig. S3**)**. No significant difference was observed between intervention delivery modalities (*P* = 0.62**) (**Supplementary Fig. S4**)**. The greatest benefits were observed among patients with permanent stomas (SMD = 2.63; *P* < 0.001) **(**Supplementary Fig. S5**)**. Nurse-led status did not significantly modify the treatment effect (*P* = 0.31) **(**Supplementary Fig. S6**)**. Intervention duration was also a significant moderator (*P* = 0.04) **(**Supplementary Fig. S7**)**, with significant improvements confined to medium-term programs (2–3 months: SMD = 1.53; 95% CI: 0.58–2.47). Funnel plot asymmetry suggested a potential for publication bias or small-study effects **(**Supplementary Fig. S8**).**

#### Impact on quality of life

**Fig. 4:**
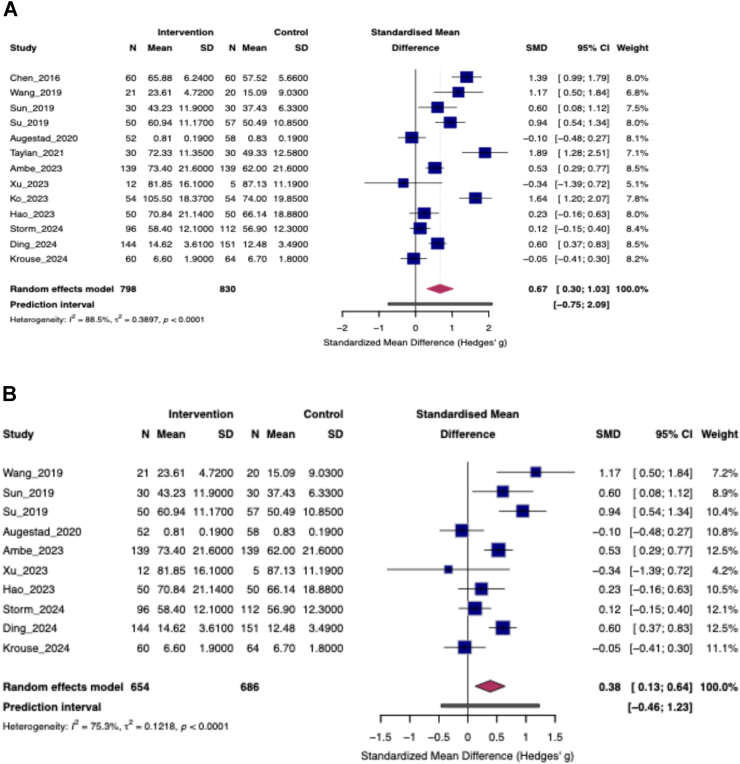
A: Meta-analysis of Digital Health Interventions for Quality of Life (QoL). Forest plot showing the overall effect of DHIs on QoL across 13 studies. Squares represent the standardized mean difference (SMD) for individual studies; diamond represents the pooled SMD; horizontal lines represent 95% CIs. SMD was calculated as Hedges' g to correct for small-sample bias. B: Sensitivity Analysis: Forest Plot Excluding Extreme Outliers. The reduction in *I*^2^ from 88.5% to 75.3% demonstrates that these trials were influential drivers of variance, though the overall positive effect of DHIs on QoL remains robust (*P* = 0.003).

A meta-analysis of 13 RCTs (*n* = 1628) ^11^^,^^12^^,^^14^^,^^15^^,^^22^, ^23^, ^25^, ^24^^,^^27^^,^^28^^,^^30^, ^31^, ^32^ was performed to evaluate the impact of DHIs on patient quality of life. The random-effects model revealed a significant improvement in QoL for the DHI group compared to standard care, with a pooled SMD of 0.67 (95% CI: 0.30–1.03; *P* = 0.0003). Statistical heterogeneity was substantial (*I*^2^ = 88.5%; τ² = 0.3897, *P* < 0.0001) as shown in Fig. 4A. A sensitivity analysis was conducted by excluding three extreme outliers.^25^^,^^31^^,^^32^ The pooled estimate remained statistically significant following the exclusion, but became more conservative (SMD = 0.38; 95% CI: 0.13–0.64; *P* = 0.003) while heterogeneity was reduced to 75.3%. These results confirm that DHIs provide a consistent, moderate benefit to QoL across the evidence base as shown in Fig. 4B. Sensitivity analysis excluding non-validated scales (SMD = 0.56; 95% CI: 0.17–0.95; *P* = 0.005; *I*^2^ = 87.8%) confirmed a significant benefit on QoL (Supplementary Fig. S9). Leave-one-out analysis further corroborated these findings and no single trial exclusion transitioned the pooled result to non-significance (SMD range: 0.58–0.74; all *P* < 0.05) **(**Supplementary Fig. S10**).**

Exploratory subgroup analysis for the QoL domain identified stoma type as a significant moderator of the treatment effect (*P* = 0.041; Supplementary Fig. S11) with the largest improvements observed in patients with permanent stomas (SMD = 1.39) compared to mixed (SMD = 0.50), urinary (SMD = 0.80), or temporary (SMD = 0.81) cohorts. Subgroup analysis identified no significant difference in QoL outcomes between economic settings (*P* = 0.082). Although the treatment effect was more pronounced in UMICs (SMD = 0.95; 95% CI: 0.54–1.35) than in HICs (SMD = 0.33; 95% CI: −0.23–0.89) (Supplementary Fig. S12).

Subgroup analysis revealed that delivery modality did not significantly modify the treatment effect on QoL (*P* = 0.86) with comparable improvements observed across both digital platforms (SMD = 0.64; 95% CI: 0.15–1.13) and tele-support program (SMD = 0.71; 95% CI: 0.11–1.31) (Supplementary Fig. S13). Nurse involvement did not significantly moderate QoL (*P* = 0.84) as comparable effect sizes were observed in both nurse-led interventions (SMD = 0.64; 95% CI: 0.22–1.07) and those without nurse involvement (SMD = 0.75; 95% CI: −0.13–1.62) (Supplementary Fig. S14). Scale type was not a significant moderator (*P* = 0.13) **(**Supplementary Fig. S15**)**. Significant QoL improvements were isolated to stoma-specific scales (SMD = 0.86; 95% CI: 0.38–1.35) and were not observed with generic instruments. Visual inspection of the funnel plot demonstrated a degree of asymmetry, indicating potential publication bias for the QoL outcome (Supplementary Fig. S16).

#### Secondary outcomes: impact on patient satisfaction

**Fig. 5:**
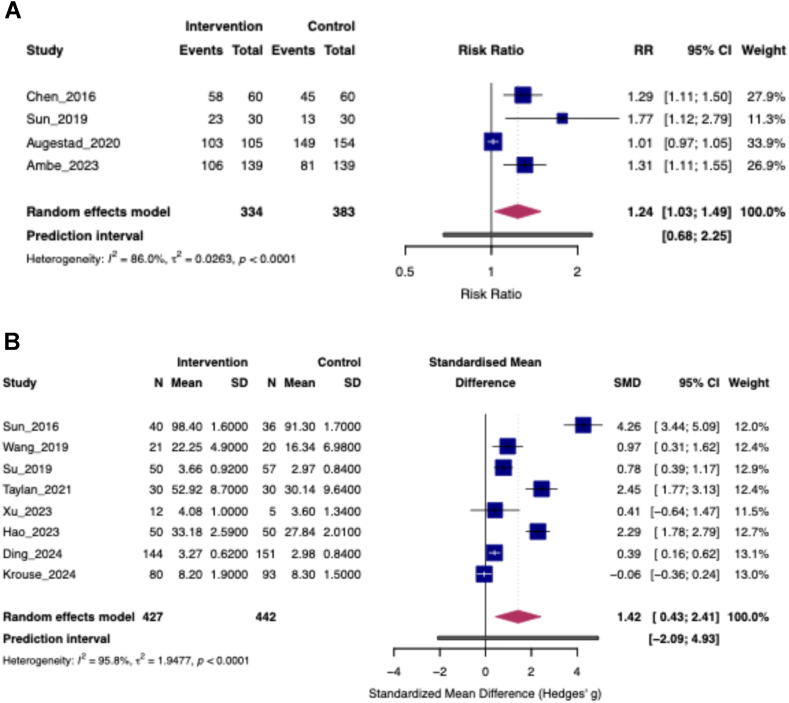
A: Forest Plots for Patient Satisfaction: Binary Outcomes. Binary outcomes: Forest plot illustrating the pooled risk ratio (k=4) for reported satisfaction in patients with an ostomy. Red diamonds represent pooled effect sizes using a random-effects model; horizontal bars indicate 95% CIs. B: Forest Plots for Patient Satisfaction: Continuous Outcomes. Continuous outcomes: Forest plot illustrating the standardized mean difference (*k* = 8) for satisfaction scores. Red diamonds represent pooled effect sizes using a random-effects model; horizontal bars indicate 95% CIs. CI, confidence interval; RR, risk ratio; SMD, standardized mean difference. (For interpretation of the references to colour in this figure legend, the reader is referred to the web version of this article.)

Patient satisfaction was synthesized across 12 RCTs using both binary and continuous outcomes. In studies reporting binary data (*k =* 4, *n* = 717), DHIs significantly increased the likelihood of patient satisfaction (RR = 1.24; 95% CI: 1.03–1.49; *P* = 0.026) (Fig. 5A). In studies using continuous satisfaction scales (*k =* 8, *n* = 869), a large positive effect was observed (SMD = 1.42; 95% CI: 0.43–2.41; *P* = 0.005) (Fig. 5B). However, continuous outcomes exhibited extreme heterogeneity (*I*^*2*^ = 95.8%), which was largely explained by economic context in subgroup analysis.

The binary significance was sensitive to single-study withdrawal (*P* = 0.07) (Supplementary Fig. S17). Continuous results remained robust after outlier exclusion (SMD = 0.46; *P* = 0.02) (Supplementary Fig. S18). No significant moderating effect of economic status was observed for binary satisfaction outcomes (*P* = 0.266) **(**Supplementary Fig. S19**)**. Economic status significantly moderated continuous satisfaction (*P* = 0.002) with significant benefits isolated to UMICs (SMD = 1.8258; 95% CI: 0.6874–2.9642) compared to HICs (SMD = −0.0236; 95% CI: −0.3112–0.2640) **(**Supplementary Fig. S20**)**. Delivery modality significantly moderated the binary outcome **(**Supplementary Fig. S21**)** and did not significantly differ for the continuous outcome **(**Supplementary Fig. S22**)**.

#### Impact on postoperative complications

**Fig. 6:**
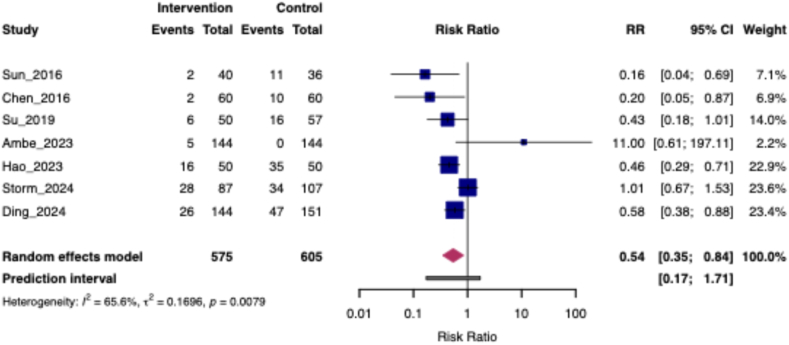
Forest Plot of the Effect of Digital Health Interventions on Postoperative Complications. The forest plot displays the primary analysis for postoperative complications. Weights are assigned using the random-effects model (REML method). The red diamond represents the pooled risk ratio, and horizontal bars represent 95% CIs. The red dashed line indicates the 95% prediction interval. CI, confidence interval; RR, risk ratio. (For interpretation of the references to colour in this figure legend, the reader is referred to the web version of this article.)

The impact of DHIs on stoma-related complications was evaluated across 7 RCTs (*n* = 1180).^12^^,^^14^^,^^15^^,^^22^^,^^23^^,^^25^^,^^26^^,^^30^ Meta-analysis using a random-effects model showed that participants in the DHI group had a 46% lower risk of experiencing complications compared to the control group (RR, 0.54; 95% CI: 0.35–0.84; *P* = 0.006; τ^2^ = 0.1696; *I*^2^ = 65.6%; Fig. 6). Sensitivity analysis excluding Ambe (2023) confirmed a robust reduction in risk (RR = 0.5091; 95% CI: 0.3268–0.7931; *P* = 0.002; τ^2^ = 0.1674) **(**Supplementary Fig. S23**)**. Leave-one-out analysis demonstrated that the overall effect was predominantly stable, though the omission of Hao et al.^22^ marginally attenuated the significance (*P* = 0.0529) **(**Supplementary Fig. S24**)**. Notably, the exclusion of Storm et al.^30^ significantly reduced heterogeneity (*I*^2^ = 43.9%; *P* < 0.0001). Subgroup analyses indicated that the effect was not significantly moderated by economic status (*P* = 0.174) (Supplementary Fig. S25) or stoma type (*P* = 0.5220) **(**Supplementary Fig. S26**)**. However, the protective effect was statistically significant in UMICs (RR = 0.4705; 95% CI: 0.3567–0.6206; τ^2^ < 0.0001) but not in HICs (RR = 2.1386; 95% CI: 0.2446–18.7004; τ^2^ = 1.7383). Visual inspection of the funnel plot suggested moderate asymmetry, potentially indicating small-study effects or reporting bias in this domain (Supplementary Fig. S27).

#### Impact on psychological well-being

**Fig. 7:**
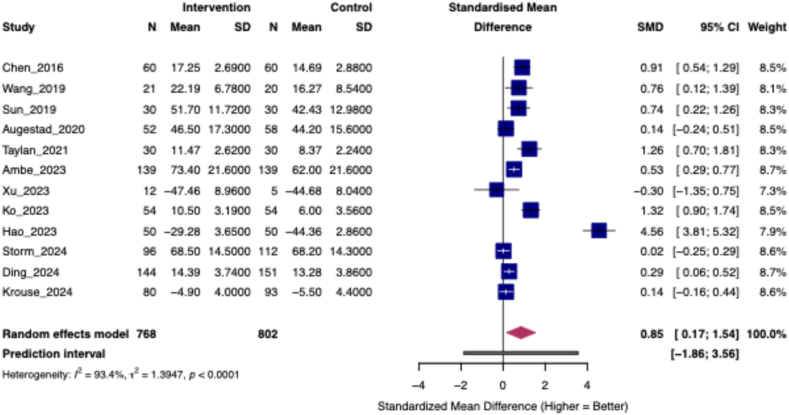
Forest Plot of Digital Health Interventions Versus Control for Psychological Well-Being. Squares represent the standardized mean difference (SMD) for individual studies; the diamond represents the pooled SMD; horizontal lines represent 95% CIs. SMD was calculated as Hedges' g. Higher SMD values indicate better psychological well-being (lower distress or higher mental health scores).

The impact of DHIs on psychological well-being was evaluated across 12 RCTs (*n* = 1570) ^12^^,^^14^^,^^15^^,^^22^^,^^24^^,^^25^^,^^27^, ^28^, ^29^, ^30^, ^31^, ^32^. Random-effects meta-analysis revealed a statistically significant improvement in psychological well-being in the DHI group compared to controls (SMD = 0.85; 95% CI: 0.17–1.54; *P* = 0.0147). Statistical heterogeneity was high (*I*^2^ = 93.4%; *P* < 0.0001; τ^2^ = 1.3947; Fig. 7). Subgroup analysis by scale type (positive vs. negative metrics) indicated no significant moderating effect (*P* = 0.5898), although a consistent and significant benefit was observed in the positive scale subgroup (SMD = 0.630; 95% CI: 0.3245–0.936) **(**Supplementary Fig. S28**)**. Similarly, subgroup analysis by economic status did not identify a significant moderator effect (*P* = 0.115) (Supplementary Fig. S29). However, the treatment effect achieved significance in trials conducted in UMICs (SMD = 1.3989; 95% CI: 0.1684–2.6294) but failed to reach significance in high-income countries (SMD = 0.3525; 95% CI: −0.0711–0.7760). Funnel plot asymmetry is shown in Supplementary Fig. S30.

#### Health care utilization and intervention feasibility

Exploratory analyses were conducted for unplanned readmissions, intervention adherence/retention, and health care utilization. No statistically significant differences were observed between the DHI and control groups across these metrics. The risk of unplanned readmissions (*k =* 3, *n* = 577) did not differ significantly between groups (RR, 0.96; 95% CI: 0.32–2.85; *P = 0.942*; *I*^2^ = 71.5%) (Supplementary Fig. S31), with conflicting directions identified between individual trials. Similarly, no significant difference was found regarding participant adherence or retention rates (*k =* 5, *n* = 910; RR = 0.96; 95% CI: 0.84–1.10; *P* = 0.537; *I*^2^ = 73.2%) (Supplementary Fig. S32). Data from a single trial (*n* = 23), health care utilization (frequency of outpatient visits) was comparable between groups (SMD = −0.48; 95% CI: −1.38–0.42; *P* = 0.299) (Supplementary Fig. S33).

### Publication bias assessment

Funnel plot assessments suggested potential small-study effects for psychological well-being and self-management competence, characterized by right-sided asymmetry favoring positive intervention effects. In contrast, plots for stoma-related complications remained largely symmetrical, while the scattered distribution observed for quality of life (*I*^2^ = 88.5%) likely reflects substantial clinical heterogeneity rather than systematic reporting bias. Overall, these findings indicate that while clinical outcomes remain robust, psychosocial and self-care estimates may be moderately influenced by reporting bias.

### Certainty of evidence (GRADE assessment)

The certainty of evidence for all outcomes was evaluated using the GRADE framework. Overall, the evidence certainty ranged from moderate to very low. The certainty of evidence for both self-management competence (SMD = 0.96) and quality of life (SMD = 0.67) was rated as low. These outcomes were downgraded primarily due to substantial statistical inconsistency (*I*^2^ > 75%) and a high risk of bias relative to the inability to blind participants and personnel. A common challenge in digital health interventions. The strongest evidence was found for stoma-related complications, which was rated as moderate certainty. DHIs significantly reduced the risk of complications by 46% (RR = 0.54; 95% CI: 0.35–0.84), with less heterogeneity than other domains. Conversely, psychological well-being was rated as low certainty, and patient satisfaction (SMD = 1.42) was rated as very low certainty due to extreme heterogeneity and the influence of statistical outliers. Finally, the evidence regarding unplanned readmissions was rated as low certainty; while a trend toward risk reduction was observed (RR = 0.87), the results did not reach statistical significance (Table 2).

## Discussion

### Main findings

This meta-analysis confirms that DHIs are a transformative adjunct to traditional stoma care. Their use significantly enhancing patients’ capacity for independent management while improving QoL in ostomy care. The most clinically robust evidence suggests that digital tools are highly effective in reducing stoma-related complications with a moderate degree of certainty. Previous reviews have hinted at the potential of mHealth in surgical recovery.^11^ Our study quantifies a significant 46% reduction in stoma-related complications. The perceived impact on psychological well-being and clinical outcomes like readmission remains subject to low certainty due to high heterogeneity. The overall findings position DHIs as a safe, feasible, and highly satisfactory resource for empowering patients with an ostomy.^4^^,^^14^^,^^33^

### Mechanisms of self-management and stoma type

Self-management competence emerged as the domain with the most profound improvement. Our findings suggest that DHIs act as a critical bridge during the acute recovery phase where information needs are highest and traditional face-to-face support may be intermittent. A critical observation in our study was the marked difference between self-efficacy and behavioral self-care. This finding aligns with Bandura's Social Cognitive Theory, which posits that confidence often precedes behavioral mastery.^34^ The significantly larger effect observed for self-efficacy compared to behavioral self-care ability suggests that current digital tools are perhaps more effective at shifting a patient's “confidence mindset” and perceived agency than at changing physical pouching behaviors.^7^ While the effect on self-efficacy appeared large, our sensitivity analysis indicates this result may be influenced by measurement bias and statistical significance was attenuated when only validated instruments were included.

The finding that patients with permanent stomas derived the greatest benefit from digital tools is particularly noteworthy. Unlike those with temporary diversions who may view stoma care as a transient burden, permanent ostomates must navigate lifelong psychosocial and physical adjustments. Continuous, on-demand access to digital troubleshooting and peer-support modules likely facilitates a more sustained level of adjustment for this cohort.^12^^,^^24^ This finding mirrors meta-analyses in other chronic conditions such as diabetes and heart failure where digital tools effectively improve “self-care confidence” but show more modest impacts on objective clinical behaviors.^35^

### Economic context as a moderator of intervention effectiveness

An important finding of this review is the disparity in intervention efficacy between economic settings. The impact of DHIs was consistently more pronounced in UMICs compared to HICs. The pronounced benefit observed in UMICs compared to HICs supports the Digital Leapfrog hypothesis.^36^ In HICs, specialized stoma care is often already optimized through established nurse-led networks therefore creating a ceiling effect for digital adjuncts. In contrast in UMICs, DHIs likely bypass structural barriers to care such as distance to tertiary centers and a shortage of specialized enterostomal therapists. They offer a high-value alternative to basic standard care.^10^^,^^12^^,^^13^ This finding suggests that DHIs are not merely supplements but are critical equity tools in resource-limited health care systems.

### Clinical impact on stoma complications and QoL

One of the most clinically significant results is the reduction in stoma-related complications such as peristomal dermatitis and leakage. These issues are the primary drivers of psychological distress and costly hospital readmissions.^1^^,^^8^^,^^37^ In standard clinical practice peristomal skin complications occur in 25%–75% of patients and are the primary drivers of psychological distress.^11^^,^^29^^,^^38^ Our findings suggest that DHIs provide the real-time troubleshooting necessary to combat user fatigue and early-stage complications.^10^

However, the smaller effect size for QoL and its low GRADE certainty reflect the inherent complexity of post-ostomy adjustment. Unlike self-care skills which can be acquired rapidly, QoL is influenced by deep-seated concerns regarding body image and social reintegration.^5^ Our results align with a previous study^39^ noted that can improve care coordination but it cannot fully replace the psychosocial human touch required for long-term emotional adjustment.^39^^,^^40^

### Health care utilization

A recurring critique of digital interventions is the potential for digital fatigue leading to high attrition.^41^ Our exploratory findings of equivalent retention rates between DHI and control groups provide strong evidence for the feasibility and acceptability of digital stoma care. Furthermore, the lack of an increase in readmissions or health care visits addresses the hyper-vigilance concern. It suggests that DHIs satisfy patient needs without inducing unnecessary healthcare-seeking behavior.^16^^,^^30^ It helps maintain high retention rates without overwhelming hospital resources.

### Limitations

This meta-analysis has several important limitations that must be considered when interpreting its findings. First, the substantial statistical heterogeneity observed across most outcomes reflects the inherent diversity of digital health as an intervention class. The included studies varied widely in platform sophistication, human support components, and theoretical underpinnings, complicating direct comparison. Second, the inability to blind participants to a digital intervention introduces a high risk of performance bias, which is a systematic methodological constraint in this field and contributes to the downgrading of evidence certainty. Third, visual asymmetry in funnel plots for key outcomes suggests the potential for small-study effects or publication bias. Finally, the predominance of short-term follow-up data limits our understanding of whether the observed benefits on self-management and quality of life are sustained beyond the initial postoperative period.

### Clinical and research implications

For Clinicians: DHIs should be integrated into the Enhanced Recovery After Surgery (ERAS) pathways for ostomates. Importantly for those with permanent stomas who require a lifelong learning resource.

For Researchers: There is an urgent need for the adoption of a Core Outcome Set (COS). Future trials should prioritize longitudinal data and utilize the Consolidated Standards of Reporting Trials-EHEALTH (CONSORT-EHEALTH) checklist to improve reporting transparency and reduce the unexplained heterogeneity observed in this meta-analysis.

## Conclusions

This systematic review and meta-analysis demonstrate that DHIs are effective, feasible, and highly satisfactory tools for enhancing stoma care. They significantly improve self-management competence and quality of life while reducing the risk of stoma-related complications. The magnitude of benefit is most pronounced in upper-middle-income countries and among patients with permanent ostomies. It highlighted DHIs’ role as both an equity tool and a lifelong support resource. Despite limitations related to heterogeneity and evidence certainty, these findings strongly advocate for the integration of digital tools into standard postoperative pathways as a core component of modern, patient-centered ostomy care.

## CRediT authorship contribution statement

Donglin Wang and Na Zhou have made significant contributions to the conceptualization, methodology, data analysis, and interpretation, as well as the writing, editing, and visualization of the manuscript. Yingjie Zhang and Jianjuan Dai critically revised the statistical methods. Yingjie Zhang was the supervisor and administrator of the project. Na Zhou made substantial contributions to the editing and revision of the manuscript. All authors read and approved the final manuscript.

## Ethics statement

Not required.

## Data availability statement

The authors confirm that the data supporting the findings of this study are available within the article and its supplementary materials.

## Declaration of generative AI and AI-assisted technologies in the writing process

During the preparation of this work, the authors used Gemini 3.0 Pro solely to improve language readability and correct grammar. After using this tool, the authors reviewed and edited the content as needed and take full responsibility for the content of the publication.

## Funding

This study received no external funding.

## Declaration of competing interest

The authors declare no conflict of interest.
