# Supplementary material for: Effects of digital health interventions on self-care and quality of life in patients with an ostomy: A systematic review and meta-analysis
Source: Asia Pac J Oncol Nurs. 2026 Apr 16;13:100955. doi: 10.1016/j.apjon.2026.100955 (PMC13273889; doi:10.1016/j.apjon.2026.100955)
Supplement: Multimedia component 1 [file mmc1.docx]

**Supplmentary Tables**

**Supplementary Table S1: Search Strategy** and **Conceptual Search Terms Grouped by PICO Elements**

| **PICO Element** | **Keywords/Free-text terms** | **Controlled Vocabulary (e.g., MeSH, Emtree, CINAHL Headings)** | **Corresponding Chinese Keywords (for CNKI/Wanfang)** |
| --- | --- | --- | --- |
| **P: Population** | ostomy, stoma, colostomy, ileostomy, urostomy | “ostomy”, “stoma”, “colostomy”, “ileostomy”, “urostomy” (and their specific equivalents) | 造瘘, 造口, 结肠造口, 回肠造口, 尿路造口 |
| **I: Intervention** | mHealth, telehealth, eHealth, e-health, digital*, app, remote monitoring, virtual care, online support, mobile phone, smartphone, web-based, internet-based, artificial intelligence, AI, wearable device* | “digital health”, “telemedicine”, “mobile applications”, “eHealth”, “wearable electronic devices”, “artificial intelligence” (and their specific equivalents) | 数字健康, 远程医疗, 移动应用, 电子健康, 可穿戴设备, 移动健康, 远程监护, 虚拟护理, 在线支持, 智能手机, 互联网, 人工智能, AI |
| **O: Outcomes** | self-management, self-efficacy, patient education, patient satisfaction, adherence, readmission, psychological adjustment, anxiety, depression, body image, stoma complication*, peristomal skin, QoL | “self-care”, “quality of life”, “anxiety”, “depression”, “body image” (and their specific equivalents) | 自我护理, 生活质量, 自我管理, 自我效能, 患者教育, 患者满意度, 依从性, 再入院, 心理调适, 焦虑, 抑郁, 身体形象, 造口并发症, 造口周围皮肤 |

**Supplementary Table S2: PubMed (Medline) Search Strategy**

| **#** | **Search Term** |
| --- | --- |
| 1 | (“ostomy”[MeSH Terms] OR “stoma”[MeSH Terms] OR “colostomy”[MeSH Terms] OR “ileostomy”[MeSH Terms] OR “urostomy”[MeSH Terms] OR ostomy[tiab] OR stoma[tiab] OR colostomy[tiab] OR ileostomy[tiab] OR urostomy[tiab]) |
| 2 | (“digital health”[MeSH Terms] OR “telemedicine”[MeSH Terms] OR “mobile applications”[MeSH Terms] OR “eHealth”[MeSH Terms] OR “wearable electronic devices”[MeSH Terms] OR mHealth[tiab] OR telehealth[tiab] OR eHealth[tiab] OR “e-health”[tiab] OR digital*[tiab] OR app[tiab] OR “remote monitoring”[tiab] OR “virtual care”[tiab] OR “online support”[tiab] OR “mobile phone”[tiab] OR “smartphone”[tiab] OR “web based”[tiab] OR “internet based”[tiab] OR “artificial intelligence”[MeSH Terms] OR AI[tiab] OR “wearable device*”[tiab]) |
| 3 | (“self care”[MeSH Terms] OR “quality of life”[MeSH Terms] OR “self-management”[tiab] OR “self efficacy”[tiab] OR “patient education”[tiab] OR “patient satisfaction”[tiab] OR adherence[tiab] OR readmission[tiab] OR “psychological adjustment”[tiab] OR anxiety[tiab] OR depression[tiab] OR “body image”[tiab] OR “stoma complication*”[tiab] OR “peristomal skin”[tiab] OR QoL[tiab]) |
| 4 | #1 AND #2 AND #3 |
| 5 | Filter: Randomized Controlled Trial (RCT) |
| 6 | Filter: Publication date from 2015 to 2025 |
| 7 | Filter: Language (English) |

**Supplementary Table S3: Embase Search Strategy**

| **#** | **Search Term** |
| --- | --- |
| 1 | (‘ostomy’/exp OR ‘stoma’/exp OR ‘colostomy’/exp OR ‘ileostomy’/exp OR ‘urostomy’/exp OR ostomy:ab,ti OR stoma:ab,ti OR colostomy:ab,ti OR ileostomy:ab,ti OR urostomy:ab,ti) |
| 2 | (‘digital health’/exp OR ‘telemedicine’/exp OR ‘mobile application’/exp OR ‘eHealth’/exp OR ‘wearable electronic device’/exp OR mHealth: ab,ti OR telehealth:ab,ti OR eHealth:ab,ti OR ‘e-health’:ab,ti OR digital*:ab,ti OR app:ab,ti OR ‘remote monitoring’:ab,ti OR ‘virtual care’:ab,ti OR ‘online support’:ab,ti OR ‘mobile phone’:ab,ti OR smartphone:ab,ti OR ‘web based’:ab,ti OR ‘internet based’:ab,ti OR ‘artificial intelligence’/exp OR AI:ab,ti OR ‘wearable device*’:ab,ti) |
| 3 | (‘self care’/exp OR ‘quality of life’/exp OR ‘self management’:ab,ti OR ‘self efficacy’:ab,ti OR ‘patient education’:ab,ti OR ‘patient satisfaction’:ab,ti OR adherence:ab,ti OR readmission:ab,ti OR ‘psychological adjustment’:ab,ti OR anxiety:ab,ti OR depression:ab,ti OR ‘body image’:ab,ti OR ‘stoma complication*’:ab,ti OR ‘peristomal skin’:ab,ti OR QoL:ab,ti) |
| 4 | #1 AND #2 AND #3 |
| 5 | Filter: Randomized controlled trial |
| 6 | Filter: Publication date from 2015 to 2025 |
| 7 | Filter: Language (English) |

**Supplementary Table S4: Cochrane Central Register of Controlled Trials (CENTRAL) Search Strategy**

| **#** | **Search Term** |
| --- | --- |
| 1 | (ostomy OR stoma OR colostomy OR ileostomy OR urostomy):ti,ab |
| 3 | (“self care” OR “quality of life” OR “self-management” OR “self efficacy” OR “patient education” OR “patient satisfaction” OR adherence OR readmission OR “psychological adjustment” OR anxiety OR depression OR “body image” OR “stoma complication*” OR “peristomal skin” OR QoL):ti,ab |
| 4 | #1 AND #2 AND #3 |
| 5 | Filter: Publication date from 2015 to 2025 |
| 6 | Filter: Language (English) |

**Supplementary Table S5: CINAHL (Cumulative Index to Nursing and Allied Health Literature) Search Strategy**

| **#** | **Search Term** |
| --- | --- |
| 1 | (MH “Ostomy+”) OR (MH “Stoma+”) OR (MH “Colostomy+”) OR (MH “Ileostomy+”) OR (MH “Urostomy+”) OR (ostomy OR stoma OR colostomy OR ileostomy OR urostomy) in TI,AB |
| 2 | (MH “Digital Health”) OR (MH “Telemedicine”) OR (MH “Mobile Applications”) OR (MH “eHealth”) OR (MH “Wearable Electronic Devices”) OR (mHealth OR telehealth OR eHealth OR “e-health” OR digital* OR app OR “remote monitoring” OR “virtual care” OR “online support” OR “mobile phone” OR smartphone OR “web based” OR “internet based” OR “artificial intelligence” OR AI OR “wearable device*”) in TI,AB |
| 3 | (MH “Self Care+”) OR (MH “Quality of Life+”) OR (“self-management” OR “self efficacy” OR “patient education” OR “patient satisfaction” OR adherence OR readmission OR “psychological adjustment” OR anxiety OR depression OR “body image” OR “stoma complication*” OR “peristomal skin” OR QoL) in TI,AB |
| 4 | #1 AND #2 AND #3 |
| 5 | Limiters: Randomized Controlled Trial |
| 6 | Limiters: Publication Date: 2015 to 2025 |
| 7 | Limiters: English Language |

**Supplementary Table S6: Chinese Databases (CNKI, Wanfang Data) Search Strategy**

.

| **#** | **Search Term (Chinese Characters)** | **Search Term (English Translation)** |
| --- | --- | --- |
| 1 | (造瘘 OR 造口 OR 结肠造口 OR 回肠造口 OR 尿路造口) | (Ostomy OR Stoma OR Colostomy OR Ileostomy OR Urostomy) |
| 2 | (数字健康 OR 远程医疗 OR 移动应用 OR 电子健康 OR 可穿戴设备 OR 移动健康 OR 远程监护 OR 虚拟护理 OR 在线支持 OR 智能手机 OR 互联网 OR 人工智能 OR AI) | (Digital health OR Telemedicine OR Mobile applications OR eHealth OR Wearable devices OR mHealth OR Remote monitoring OR Virtual care OR Online support OR Smartphone OR Internet OR Artificial Intelligence OR AI) |
| 3 | (自我护理 OR 生活质量 OR 自我管理 OR 自我效能 OR 患者教育 OR 患者满意度 OR 依从性 OR 再入院 OR 心理调适 OR 焦虑 OR 抑郁 OR 身体形象 OR 造口并发症 OR 造口周围皮肤) | (Self-care OR Quality of life OR Self-management OR Self-efficacy OR Patient education OR Patient satisfaction OR Adherence OR Readmission OR Psychological adjustment OR Anxiety OR Depression OR Body image OR Stoma complications OR Peristomal skin) |
| 4 | #1 AND #2 AND #3 | Combined terms |
| 5 | Filter: | Randomized Controlled Trial (RCT) (where applicable) |
| 6 | Filter: | Publication date from 2015 to 2025 |
| 7 | Filter: | Language (Chinese) |

For these databases, the search terms were entered in Chinese characters, fully adapted to their specific platform syntax. The filters for RCTs and publication dates were applied where available.
