# Supplementary material for: Effects of digital health interventions on self-care and quality of life in patients with an ostomy: A systematic review and meta-analysis
Source: Asia Pac J Oncol Nurs. 2026 Apr 16;13:100955. doi: 10.1016/j.apjon.2026.100955 (PMC13273889; doi:10.1016/j.apjon.2026.100955)
Supplement: Multimedia component 2 [file mmc2.docx]

**Supplementary Figures**

**Self-management Competence**

**Fig. S1:**

Supplementary Fig. S1: Sensitivity Analysis excluding Studies with Non-Validated Scales for Self-Management

Note: This forest plot illustrates the meta-analytic results for self-management following the exclusion of trials utilizing non-validated assessment tools (e.g., Chen [2016] and Wang et al [2019]). The statistical significance of certain sub-domains, particularly self-efficacy in patients with an ostomy, was diminished upon exclusion of these trials. CI indicates confidence interval; SMD, standardized mean difference.

**Fig. S2:**

Supplementary Fig. S2: Leave-One-Out Sensitivity Analysis of Self-management Competence

Note: Forest plot illustrating the recalculated pooled effect after systematic exclusion of each individual study. Each row represents a meta-analysis omitting the listed trial. Data points indicate standardized mean differences; horizontal bars indicate 95% CIs. The vertical dashed line represents the null effect. CI indicates confidence interval; SMD, standardized mean difference**.**

**Fig. S3:**

Supplementary Fig. S3: Subgroup Analysis of Self-Management Competence by Economic Setting.

Note: Analysis stratified by High-Income Countries (HIC) and Upper-Middle-Income Countries (UMIC). The test for subgroup differences indicates that economic context is a significant moderator of intervention efficacy (*P*=0.007). SMD, standardized mean difference; CI, confidence interval.

**Fig. S4:**

Supplementary Fig. S4: Subgroup analysis of self-management competence by intervention modality.

Note: Analysis compares purely digital platforms against tele-support interventions. No significant difference was observed between modalities in the random-effects model (*P*=0.62).

**Fig. S5:**

Supplementary Fig. S5. Subgroup analysis of self-management competence by stoma type.

Note: Analysis stratified by mixed, urinary, temporary, and permanent stomas. Patients with permanent stomas showed a significantly larger improvement in self-management competence compared to other groups (*P*<0.001).

**Fig. S6:**

Supplementary Fig. S6. Subgroup Analysis of Self-Management Competence by Nurse-Led Delivery.

Note: Compares interventions led by nursing staff against those that were not nurse-led. The difference between subgroups was not statistically significant (*P*=0.31).

**Fig. S7:**

Supplementary Fig. S7. Subgroup Analysis of Self-management Competence by Intervention Duration.

Note: Interventions were categorized as short-term or long-term. Shorter interventions were associated with significantly higher standardized mean differences than longer-duration programs (*P*=0.002).

**Fig. S8:**

Supplementary Fig. S8. Funnel Plot for Self-Management Competence.

Note: Standard error is plotted against the standardized mean difference. The observed asymmetry suggests the potential presence of publication bias or disproportionate influence from small studies with large effect sizes.

**Quality of Life**

**Fig. S9:**

Supplementary Fig. S9: Sensitivity analysis excluding Studies with Non-Validated Scales for QoL

Note: This forest plot illustrates the robustness of the quality-of-life estimate following the simultaneous exclusion of Chen (2016) and Wang et al (2019). The pooled effect of digital health interventions remains statistically significant, regardless of the inclusion of non-validated assessment tools. This confirms that the observed benefits for patients with an ostomy are not driven by measurement bias. CI indicates confidence interval; SMD, standardized mean difference.

**Fig. S10:**

Supplementary Fig. S10. Leave-one-out sensitivity analysis for quality-of-life (QoL) outcomes.

Note: This forest plot presents the results of an iterative exclusion analysis to evaluate the stability of the pooled estimate. The consistent statistical significance observed throughout all iterations demonstrates that the overall quality-of-life benefit in patients with an ostomy is robust and not disproportionately driven by any single trial. CI indicates confidence interval; SMD, standardized mean difference.

**Fig. S11:**

Supplementary Fig. S11. Subgroup analysis of quality of life by Stoma Type.

Note: Significant differences were observed based on stoma type (*P*=0.041) with permanent stoma patients showing the most substantial improvement in QoL following the intervention.

**Fig. S12:**

Supplementary Fig. S12. Subgroup Analysis of Quality of Life by Economic Context.

Note: Comparison between High-Income Countries (HIC) and Upper-Middle-Income Countries (UMIC). While UMIC studies showed a larger effect size, the subgroup difference was not significant (*P*=0.082). SMD, standardized mean difference; CI, confidence interval.

**Fig. S13:**

Supplementary Fig. S13. Subgroup Analysis of Quality of Life by Intervention Modality.

Note: Comparison of purely digital platforms versus tele-support programs. The results suggest the delivery modality does not significantly influence QoL outcomes (*P*=0.862).

**Fig. S14:**

Supplementary Fig. S14. Subgroup analysis of quality of life by nurse involvement.

Note: Compares studies with nurse-led interventions against those without. Digital health intervention efficacy on QoL was consistent regardless of nurse-led status (*P*=0.838)

**Fig. S15:**

Supplementary Fig. S15. Subgroup analysis of quality of life by scale type.

Note: Analysis stratified by disease-specific scales versus generic quality-of-life instruments. While specific scales showed a higher point estimate, the difference between groups was not statistically significant (*P*=0.129).

**Fig. S16:**

Supplementary Fig. S16. Funnel Plot for Quality of Life.

Note: Plotted standard error against the standardized mean difference. The distribution suggests moderate asymmetry, reflecting potential small-study effects or publication bias across the included trials.

**Patient Satisfaction**

**Fig. S17:**

Supplementary Fig. S17. Forest Plot of Sensitivity Analysis for Patient Satisfaction (Binary Outcome)

Note: The forest plot displays the pooled risk ratio (RR) for patient satisfaction after the exclusion of one study (Sun et al, 2019). Weights were calculated using the random-effects model (inverse variance method). The red diamond represents the pooled RR, and the horizontal bars represent the 95% CIs. The red dashed line (prediction interval) indicates the range in which the effect size of a future study is expected to fall. CI indicates confidence interval; RR, risk ratio.

**Fig. S18:**

Supplementary Fig. S18. Forest Plot of Sensitivity Analysis for Patient Satisfaction (Continuous Outcome) Following Outlier Exclusion

Note: The forest plot illustrates the sensitivity analysis for patient satisfaction measured as continuous data, excluding extreme outliers to address high heterogeneity. The effect size is expressed as Hedges’ g (bias-corrected standardized mean difference). The red diamond and horizontal bars indicate the pooled estimate and its 95% CI. The red dashed line represents the prediction interval. N indicates the number of participants; SD, standard deviation; CI, confidence interval.

**Fig. S19:**

Supplementary Fig. S19. Forest Plot of Subgroup Analysis for Patient Satisfaction (Binary Outcome) Categorized by Economic Status.

**Note:** Subgroup analysis was performed based on World Bank economic classifications. Weights are calculated using the random-effects model (inverse variance method). The test for subgroup differences shows no significant moderator effect (*P*=0.2668). The red diamond represents the pooled risk ratio, and the horizontal bars indicate 95% CIs. CI indicates confidence interval; HIC, high-income countries; RR, risk ratio; UMIC, upper-middle-income countries.

**Fig. S20:**

Supplementary Fig. S20. Forest Plot of Subgroup Analysis for Patient Satisfaction (Continuous Outcome) Categorized by Economic Status.

Note: Subgroup analysis reveals a significant moderator effect for economic status (*P*=0.002) with patient satisfaction significantly improved in UMIC settings (SMD, 1.8258; 95% CI, 0.6874–2.9642; τ^2^=1.9374) but not in HIC settings (SMD, –0.0236; 95% CI, –0.3112 to 0.2640; τ^2^=0.0000) Effect sizes are expressed as Hedges’ g (standardized mean difference). Weights are derived from the random-effects model. CI indicates confidence interval; HIC, high-income countries; SMD, standardized mean difference; UMIC, upper-middle-income countries.

**Fig. S21:**

Supplementary Fig. S21. Forest Plot of Subgroup Analysis for Patient Satisfaction (Binary Outcome) Categorized by Delivery Modality.

Note: Subgroup analysis compares digital-only platforms versus tele-support (human-led remote contact). A significant moderator effect was observed between groups (*P* < 0.0001). Weights are derived from the random-effects model using the inverse variance method. The red diamond represents the pooled risk ratio, and horizontal bars indicate 95% CIs. CI indicates confidence interval; RR, risk ratio.

**Fig. S22:**

Supplementary Fig. S22. Forest Plot of Subgroup Analysis for Patient Satisfaction (Continuous Outcome) Categorized by Delivery Modality.

**Note:** Subgroup analysis reveals no significant difference in treatment effect between digital and tele-support modalities for continuous satisfaction outcomes (*P*=0.2084). Both subgroups demonstrated individual significance, though digital interventions exhibited higher between-study variance (Digital: τ^2^=3.4188; Tele-Support: τ^2^=0.7872). Effect sizes are expressed as Hedges’ g (standardized mean difference). CI indicates confidence interval; SMD, standardized mean difference.

**Postoperative Complications**

**Fig. S23:**

Supplementary Fig. S23. Sensitivity Analysis for Postoperative Complications Excluding Ambe et al (2023).

Note: Results of the sensitivity analysis following the exclusion of an outlier study. The protective effect remains significant (*P*=0.0028). REML was utilized for τ^2^ estimation. CI indicates confidence interval; RR, risk ratio.

**Fig. S24:**

Supplementary Fig. S24. Leave-One-Out Sensitivity Analysis for Postoperative Complications.

Note: This plot illustrates the impact of omitting individual studies on the pooled risk ratio, *P*-value, τ^2^, and *I*^2^. The results remain largely consistent, confirming the stability of the primary meta-analytic estimate across the included trials. CI indicates confidence interval; PI, prediction interval; RR, risk ratio.

**Fig. S25:**

Supplementary Fig. S25. Subgroup Analysis for Postoperative Complications by Economic Status.

Note: Studies are categorized into High-Income Countries (HIC) and Upper-Middle-Income Countries (UMIC). No significant between-group difference was identified (*P*=0.174), although the UMIC subgroup exhibited lower heterogeneity (*I*^2^=8.6%). CI indicates confidence interval; RR, risk ratio.

**Fig. S26:**

**Supplementary Fig. S26**. Subgroup Analysis for Postoperative Complications by Stoma Type.

**Note:** Subgroups include Mixed, Urinary, Temporary, and Permanent stomas. The test for subgroup differences shows no significant moderator effect based on stoma classification (*P*=0.522). Weights are derived from the random-effects model. CI indicates confidence interval; RR, risk ratio.

**Fig. S27:**

Supplementary Fig. S27. Funnel Plot for the Assessment of Publication Bias in Postoperative Complications.

Note: This funnel plot illustrates the relationship between treatment effect (log risk ratio) on the x-axis and study precision (standard error) on the y-axis for postoperative complications (*k* = 7). The vertical dashed line represents the pooled effect size from the random-effects model, and the diagonal lines define the pseudo-95% confidence interval boundaries. The distribution of studies is evaluated for symmetry to identify potential publication bias or small-study effects. RR indicates risk ratio.

**Psychological Well-Being**

**Fig. S28:**

Supplementary Fig. S28. Forest Plot of Subgroup Analysis for Psychological Well-being by Scale Type.

Note: Subgroups are categorized by scale direction (positive metrics where higher scores indicate better well-being vs. negative metrics where higher scores indicate greater distress, adjusted for direction). The test for subgroup differences yielded no significant effect (*P*=0.589). Effect sizes are expressed as Hedges’ g (bias-corrected standardized mean difference). Weights are derived from the random-effects model. CI indicates confidence interval; SMD, standardized mean difference.

**Fig. S29:**

Supplementary Fig. S29. Forest Plot of Subgroup Analysis for Psychological Well-being by Economic Status.

Note: Studies were stratified into High-Income Countries (HIC) and Upper-Middle-Income Countries (UMIC). While the moderator effect was not statistically significant (*P*=0.115) the UMIC subgroup demonstrated a higher magnitude of effect with substantial heterogeneity (τ^2^=2.289; *I*^2^=95.7%. CI indicates confidence interval; SMD, standardized mean difference.

**Fig. S30:**

Supplementary Fig. S30. Funnel Plot for the Assessment of Publication Bias in Psychological Well-being.

Note: This funnel plot displays the standardized mean difference (Hedges’ g) on the x-axis against the standard error on the y-axis for *1*^2^ included studies. The vertical dashed line represents the pooled effect size. The distribution of studies is inspected for symmetry to assess potential publication bias, small-study effects, or high between-study heterogeneity. SMD indicates standardized mean difference.

**Exploratory Outcomes**

**Fig. S31:**

Supplementary Fig. S31. Forest Plot of Digital Health Interventions vs Standard Care for Unplanned Readmissions.

Note: The forest plot displays the pooled risk ratio for unplanned readmissions. A random-effects model was employed due to significant statistical heterogeneity (τ^2^=0.6806; *I*^2^=71.5%). The red diamond represents the pooled RR, and the horizontal bars indicate 95% CIs. The wide prediction interval (red dashed line) reflects the high variability in effect direction among the included studies. CI indicates confidence interval; RR, risk ratio.

**Fig. S32:**


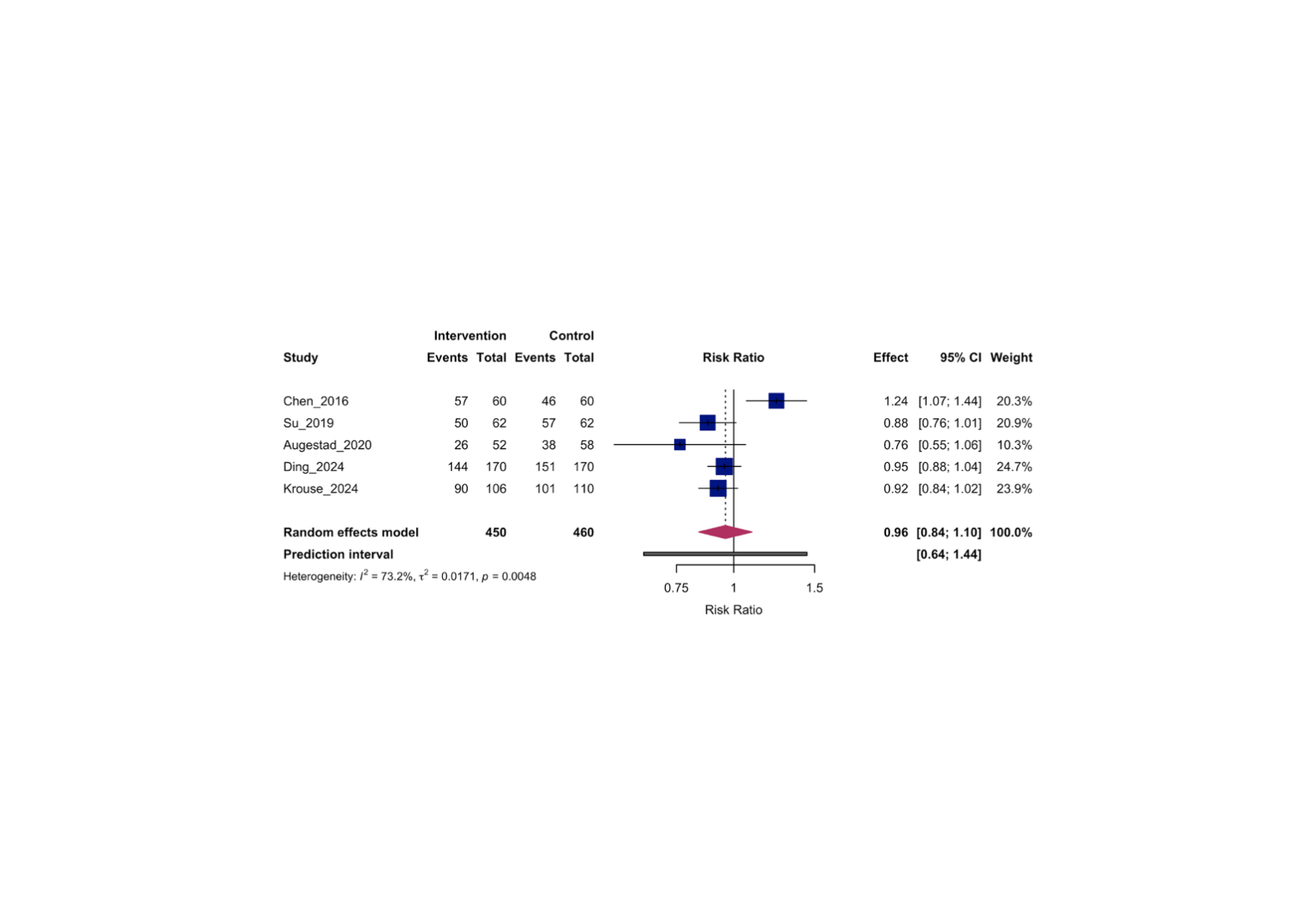


Supplementary Fig. S32. Forest Plot of Digital Health Interventions vs Standard Care for Adherence and Retention.

Note: This plot illustrates the relative risk of trial retention or intervention adherence. No significant difference was observed between DHIs and standard care (*P*=0.537). Statistical heterogeneity was substantial (*I*^2^=73.2%). Weights were calculated using the random-effects model (inverse variance method). CI indicates confidence interval; RR, risk ratio.

**Fig. S33:**

Supplementary Fig. S33. Effect of Digital Health Interventions on Healthcare Utilization (Outpatient Visits).

**Note:** Data represent the effect size from a single trial (Xu 2023) regarding the frequency of healthcare visits. The estimate is expressed as Hedges’ g (standardized mean difference). The horizontal bar indicates the 95% CI. CI indicates confidence interval; SMD, standardized mean difference.
